# Enabling X-ray fluorescence imaging for in vivo immune cell tracking

**DOI:** 10.1038/s41598-023-38536-5

**Published:** 2023-07-17

**Authors:** Theresa Staufer, Christian Körnig, Beibei Liu, Yang Liu, Clarissa Lanzloth, Oliver Schmutzler, Tanja Bedke, Andres Machicote, Wolfgang J. Parak, Neus Feliu, Lidia Bosurgi, Samuel Huber, Florian Grüner

**Affiliations:** 1 Fachbereich Physik, Universität Hamburg, 22761 Hamburg, Germany; 2 Center for Free-Electron Laser Science (CFEL), 22761 Hamburg, Germany; 3 I. Department of Medicine, University Medical Center Hamburg-Eppendorf, 20246 Hamburg, Germany; 4 Hamburg Center for Translational Immunology (HCTI), University Medical Center Hamburg-Eppendorf, 20246 Hamburg, Germany; 5 Center for Hybrid Nanostructures (CHyN), Luruper Chaussee 149, 22761 Hamburg, Germany; 6 Fraunhofer Center for Applied Nanotechnology (IAP-CAN), 20146 Hamburg, Germany; 7 Protozoa Immunology, Bernhard Nocht Institute for Tropical Medicine, 20359 Hamburg, Germany

**Keywords:** Cell migration, Imaging techniques

## Abstract

The infiltration of immune cells into sites of inflammation is one key feature of immune mediated inflammatory diseases. A detailed assessment of the in vivo dynamics of relevant cell subtypes could booster the understanding of this disease and the development of novel therapies. We show in detail how advanced X-ray fluorescence imaging enables such quantitative in vivo cell tracking, offering solutions that could pave the way beyond what other imaging modalities provide today. The key for this achievement is a detailed study of the spectral background contribution from multiple Compton scattering in a mouse-scaled object when this is scanned with a monochromatic pencil X-ray beam from a synchrotron. Under optimal conditions, the detection sensitivity is sufficient for detecting local accumulations of the labelled immune cells, hence providing experimental demonstration of in vivo immune cell tracking in mice.

## Introduction

State-of-the art Inflammatory Bowel Disease (IBD) therapies, including the treatment of Crohn’s disease (CD) and ulcerative colitis (UC), are ineffective in about 50% of patients^1^. Both diseases are chronic inflammatory disorders of the gastrointestinal tract, most likely driven by the microbiome and dysfunctional intestinal barrier, promoting a vicious cycle leading to chronic inflammation^2^. Studies on the single-cell level have shown a vast heterogeneity of involved immune cells, such as T cells, B cells and macrophages^3,4^. The role of macrophages in IBD is underlined by genetic studies and experimental colitis models^5^. Indeed, intestinal macrophages are a heterogenous population of cells, which play a key role in maintaining or reestablishing tissue homeostasis upon damage, especially by inducing resolution of inflammation^6^. Therefore, the simultaneous in vivo tracking of specific macrophage subsets is of high interest, and in combination with single-cell RNA sequencing (scRNA-seq) or flow cytometry, which both require acquisition of intestinal biopsies^7^, would help to decipher dynamics, localization and function of various macrophage subpopulations in living animals.

**Figure 1:**
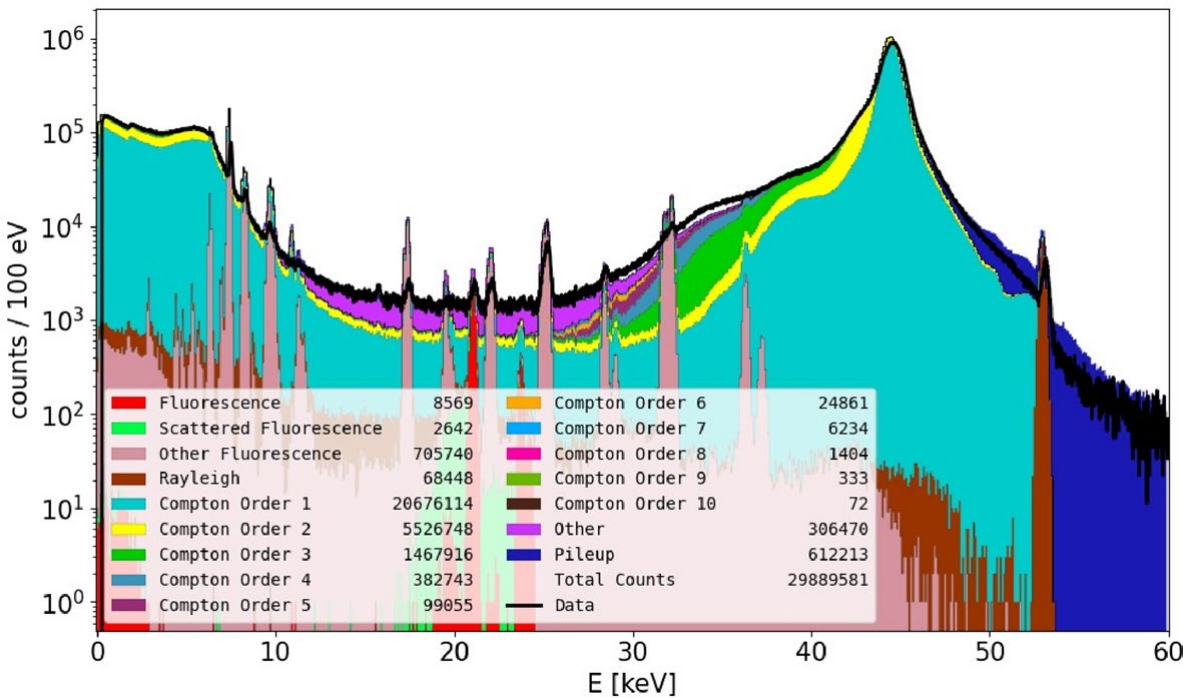
Comparison of a typical simulated and measured (black line) XFI spectrum showing good agreement in all spectral regions. The setup consisted of a Plexiglass cylinder with mouse dimensions containing an Eppendorf tube with palladium solution and an X-ray detector with 0.5 mm sensor thickness (X-123FASTSDD, Amptek Inc, MA, USA) at 60 mm distance and under an angle of 150°. One can clearly see that the majority of photons have been Compton scattered multiple times (number of Compton scattering events color coded) and the best possible signal energy range lies between 20 and 30 keV.

X-Ray Fluorescence Imaging (XFI) is a non-invasive imaging modality based on the emission of X-ray fluorescence photons from dedicated marker elements when excited by a scanning X-ray beam^8–10^. The key aspect for any experimental design of an XFI-measurement is trying to increase the detection sensitivity, which is commonly determined by the measure of the statistical significance of the expected signal (assuming that one can estimate the local marker masses). Hence, once a local marker mass is set, one can estimate the statistical significance *Z*, which can well be approximated by $Z= \frac{S}{\sqrt{B}}$, with *S* being the number of registered X-ray fluorescence photons and *B* the number of background photons in the interval centered around the fluorescence line energy with a width of typically three times the detector energy resolution. The square root of *B* is a measure for the statistical fluctuation of the background and *Z* is given in units of one standard deviation (of this background noise). Typically, *Z* should be larger than 3 or 5 to get rid of false positive signals. The number of expected fluorescence photons depends mainly on the total mass of XFI-marker atoms within the scanning pencil beam volume, the incident photon energy dependent cross-section for exciting X-ray fluorescence, and the energy dependent attenuation of the emitted fluorescence photons. These values are known or can be estimated in a straightforward way. In contrast, estimating the number *B* is non-trivial. In^11,12^ we have presented a solution for determining and minimizing *B*, however, this method only works in case of human-sized objects, i.e., when the object size is a multiple of the mean free path length of the incident photons. In this work, we focus on preclinical research with mouse-sized objects. One could expect that this spectral background *B* is very small in mouse-sized objects as this length scale is only about half of the mean free path length. However, this reasoning is too simplified. We have figured out that despite this length ratio, multiple Compton scattering plays the dominant role in determining the photon counts level *B*. For instance, when using an incident photon energy of 53 keV (as available at the P21.1 beamline of the PETRA III synchrotron at DESY, Hamburg, Germany) and iodine K-shell fluorescence, then the level of *B* is mainly set up by 5- and 6-times Compton-scattered photons. A comparison between a typical simulated and measured XFI spectrum is shown in Fig. 1, where the color-coded composition shows that the vast majority of counts have their origin in multiple Compton scattering events, that is, the inelastic scattering of an X-ray photon on an electron, leading to a decrease of the X-ray photon’s energy and a deflection with respect to its original direction.

Despite this large number of scattering events, still *B* cannot be neglected, hence defining a minimum level for *S*. It is exactly this minimum of *S* as defined by *B* which determines the sensitivity of XFI. In other words, for our goal of maximizing *Z*, we need to minimize *B* such that small values of *S* are still detectable. The strategy for this goal is to select an incident photon energy relatively far above the XFI-marker’s K-edge, because this implies that only such photons can reach the XFI-signal energy range which have undergone many Compton scattering events with decreasing probability. This reasoning also explains the way in which both a high sensitivity and a high spatial resolution can be met for XFI. For two-dimensional biodistribution “maps” of the XFI-markers, and hence the one of the labelled entities, the spatial resolution is given solely by the diameter of the scanning X-ray pencil beam. However, if the beam size is too small, then the total mass of XFI-markers inside the beam volume is also too low for yielding statistically significant signal levels against the background fluctuations. We have shown that under in vivo conditions a spatial resolution of 0.2 to 1.0 mm can be well reached while still exhibiting a sufficient sensitivity^13^. The spectral background level *B* can either be estimated by mathematical models of multiple Compton scattering or simply measured, for instance, by using a mouse phantom. This allows the optimization procedure, i.e., the finding of an optimum incident photon energy. If this energy is too high, then the fluorescence cross-section becomes too little, hence there is a clear optimum that can be found for each scanned object.

**Figure 2:**
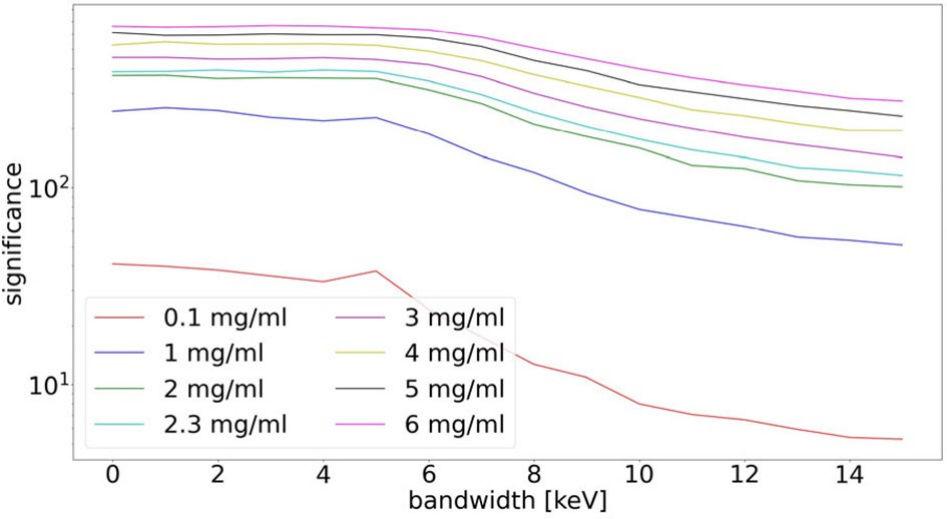
Significance of the iodine *K*_*α*_ line as a function of the bandwidth, which ranges from 0 to 15 keV (FWHM) for different marker element concentrations and a mean energy of 53 keV. While the significance value is rather constant up to a bandwidth of 5 keV, one can see a drastic reduction for higher values, especially for low concentrations, demonstrating the necessity of near monochromatic X-ray sources for high-sensitivity XFI.

So far, we have not discussed the X-ray source itself. Our experimental work is done at a synchrotron, thus with a monochromatic and zero-divergence X-ray beam of sufficient flux. The key difference between such a synchrotron-based XFI scenario and such with a conventional X-ray tube is the shape of the incident spectrum: if the spectrum is as broad as for benchtop X-ray sources, then the spectral background *B* in the signal region is very high—actually too high for reaching the levels of sensitivity presented in this work. The reason is quite clear: for a broad spectrum, photons need only a very little number of Compton scattering events in order to lower their original energy down into the signal range. Only for an almost monochromatic X-ray beam with an energy relatively far away from the signal range, *B* can be minimized since it is less probable for photons to reach the signal range at all, as demonstrated by the dependency of the significance on the bandwidth in Fig. 2.

Maximizing the sensitivity has yet another key advantage: it lowers the applied dose level. Since in this work we present a method for tracking immune cells with X-ray beams, we have taken a maximum local energy dose level of 300 mGy, as it has been reported elsewhere that T cells can perform self-repair of any radiation induced damages under such low dose levels^14,15^. A promising alternative to synchrotron-based XFI, which provides the highest level of achievable sensitivity, is the use of liquid MetalJet X-ray sources, monochromatizing the X-rays with multilayer mirrors^16^. However, thus far, the reachable incident photon energies are too low in order to fulfil the reasoning above. In addition, for low photon energies, the attenuation is quite high, hence even lowering *S*. Therefore, we want to emphasize once more that only synchrotron-like sources can provide the highest reachable XFI-sensitivity levels.

As mentioned above, XFI offers high spatial resolution, determined solely by the applied X-ray beam diameter, and at the same time high sensitivity^8–13,17^, i.e., the quantitative detection of small numbers of localized cells. Unlike commonly used modalities such as Optical Imaging (OI), e.g., Bioluminescence Imaging (BLI), XFI can be used to probe deep into tissue as hard X-ray photons are much less affected by attenuation or scattering than optical photons. Furthermore, it is explicitly suited for longitudinal studies as the non-radioactive fluorescence markers do not decay over time such as in Positron Emission-Tomography (PET) or Single-Photon Emission Computed Tomography (SPECT)^18^. In order to make biological or medical agents visible in XFI, either molecular tracers or nanoparticles can be used for labelling^8–13,17,19^. Although Magnetic Resonance Imaging (MRI) can also be performed for multi-tracking of several different markers, MRI can, unlike XFI, only be used with specific isotopes with magnetic moments and under sophisticated setups^20^. In contrast to MRI, XFI yields the information on all the different molecular or nanoparticle-based markers simultaneously in one single scan (see below)—and there is no intrinsic physical limit on a minimum XFI-scan time except for the brilliance of the X-ray source. Another key advantage of XFI is that it is a truly quantitative method, i.e., the mass of the detected labels can be directly retrieved without artefacts. Besides multi-tracking (see “Methods” section), XFI provides the unique capability of multi-scale imaging, meaning that studies on different length scales, ranging from living organisms down to individual single cells, are feasible with the same labels, offering new techniques for pharmacokinetics: after a longitudinal in vivo series of arbitrary length, individual cells can be extracted and scanned with X-ray beams focused onto sub-cellular beam sizes^17^. However, such scans of individual cells are only possible with extracted samples, i.e., ex vivo, due to the much higher applied radiation doses when beam diameters of just the cell size are used.

The pre-tests presented in the “Methods” section for the cellular uptake of the well-established iodine-based molecular contrast agent iohexol into macrophages (MHS) by inductively coupled plasma mass spectrometry (ICP-MS) show excellent agreement with the reconstructed iodine masses determined by XFI. The same applies for the multi-tracking of differently labelled MHS injected into the back of dead mice and ICP-MS measured data of a subset of these injected MHS. These pre-tests verify that XFI allows for quantitative determination of iohexol doses in biological samples. Data also revealed that the labelling with iohexol did not reduce viability, proliferation, or migration of cells (see figures in the “Methods” section). Here, we present an in vivo synchrotron-based XFI-measurement of three mice after the intraperitoneal injection of either the contrast agent iohexol alone or of iohexol labelled MHS, demonstrating the feasibility of XFI to quantitatively monitor biodistributions of labelled cells in living organisms under low dose levels.

## Results

In the preparation phase of the experiment, several phantom and in situ measurements, as well as numerical studies and analytical estimations were done in order to determine the sensitivity level and the optimal experimental setup. Among those are a simulation study^21^ and scans of a murine thyroid which does not require any labelling, as the endogenous iodine content was the target of interest^13^. Since the thyroidal iodine content of a mouse is low, the latter study demonstrates the high sensitivity of XFI when using a synchrotron as an X-ray source. The reconstructed value of 120 ng/mm^2^ already shows that tracking of labelled cells is feasible without further optimization and the step towards in vivo measurements could be taken, using the setup and techniques described in the Methods section.

**Figure 3:**
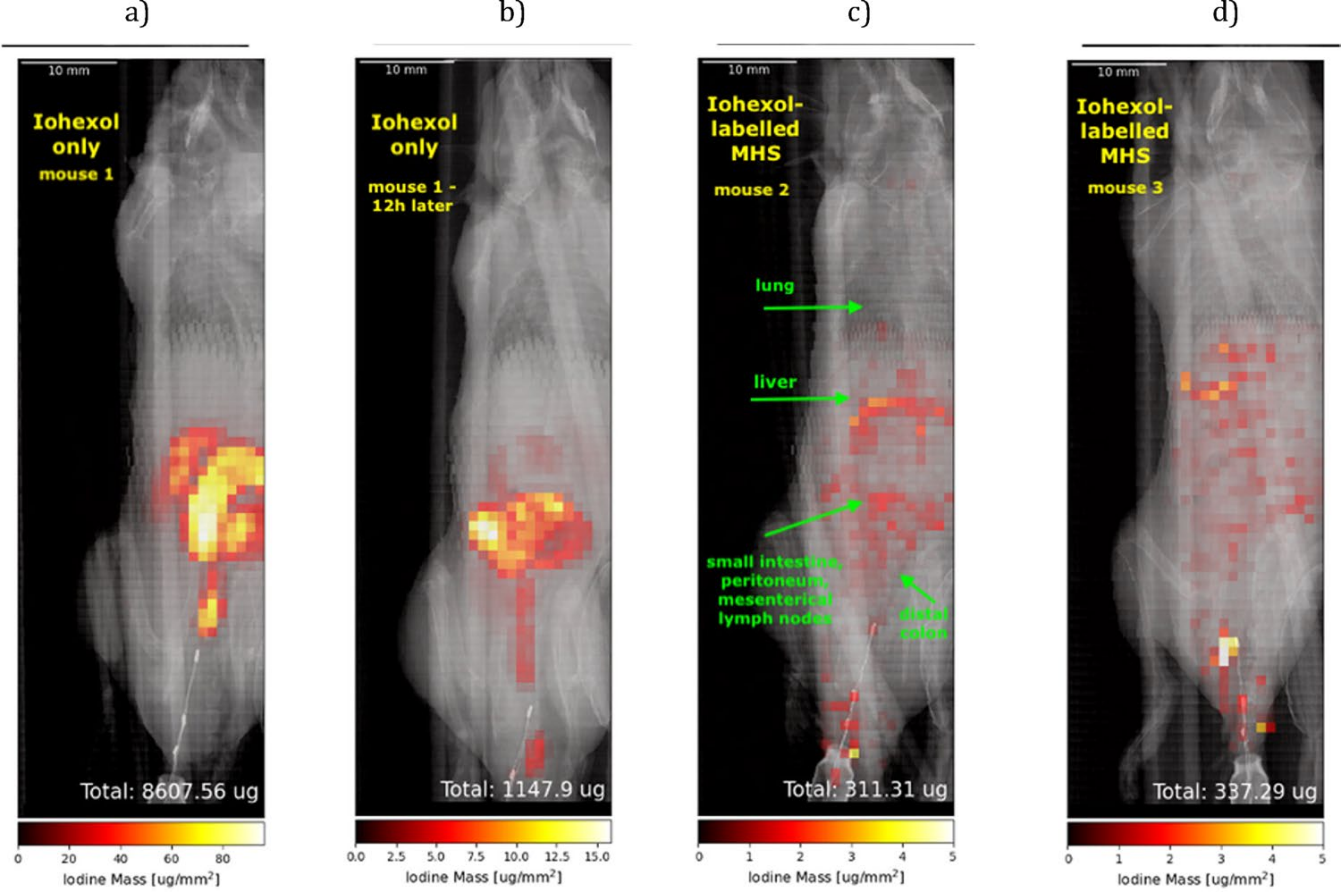
2D iodine XFI-maps overlaid with anatomical imagines obtained from X-ray transmission data for (**a**) scan of mouse 1 six hours after the injection of iohexol only, (**b**) scan of mouse 1 twelve hours after the first scan, (**c**) scan of mouse 2 six hours after the injection of MHS incubated with iohexol, (**d**) scan of mouse 3 six hours after the injection of MHS incubated with iohexol. The total reconstructed iodine mass is given in $\upmu$g and the maps were generated using python.

The reconstructed scan maps for mouse 1 (Fig. 3a and b) represent the iodine distribution 6 and 17.5 h after injection of free iohexol, which serve as a control to compare the distributions of the freely injected marker with those where the marker molecules are initially all inside the cells, thus, assessing the effect of possible exocytosis. Figure 3a and b show a significantly different distribution pattern (see the peritoneal cavity as well as the pouch of Douglas (cul-de-sac)) from the cases with the labelled cells, and their total injected free marker mass dropped down significantly. In contrast, the cases with the marker inside the cells (Fig. 3c and d) yield a reconstructed total mass of still 100% of the injected mass. This means that 100% of the transferred marker mass is visible, demonstrating the quantification ability of XFI. However, part of the iohexol may have been exocytosed during the 6 h after injection of the cells, but the total mass inside the mice is still equal to the injected dose. Mouse 2 and 3 were imaged 6 h after injection of 10 million iohexol labelled MHS each, resulting in a widespread distribution with a lower intensity in the aforementioned regions in mouse 1, and a higher expression in locations mapping to the liver, the mesenteric lymph nodes and the intestine. The total reconstructed iodine masses taken per cell result in 31.1 and 33.7 pg/cell for mouse 2 and 3, respectively, which is in excellent agreement with the ICP-MS results of 33.8 and 31.8 pg/cell.

## Discussion

Our development of the presented advanced XFI method demonstrates its feasibility to monitor in vivo the distribution of transferred MHS with sufficient sensitivity and within efficiently short full-body scan times of only 18 min per mouse, thus paving the way towards quantitative XFI-based immune cell tracking. The multi-tracking capability of XFI would be essential to gain a more detailed understanding of the pathophysiology and treatment of IBD where an imbalance of different immune cell subsets exists^22^. Not only IBD treatment but also cancer therapy based on e.g., immune check point inhibitors and CAR-T cells can potentially profit from in vivo monitoring^23^. Different to the combined method of PET/CT presented in^23^, XFI does not require the handling of radionuclides, but works with many easily accessible molecular markers or nanoparticles, and allows differentiation between differently labelled cells over long time-windows with high spatial resolution^13^ and the ability to quantify the results.

In the presented study, only macrophages were tracked, but by exploiting the multi-tracking capability of XFI, a much more detailed insight into the immune system can be gained. Since the labelling of different types of T cells is more challenging and the marker uptake lower than for macrophages^23^, there is a strong need to increase the XFI sensitivity. A major limitation currently is the small solid angle covered by the X-ray detectors which can be overcome by using much more than just a single detector in future measurements. Besides the solid angle, also the sensor thickness has to be increased in order to enhance the detection sensitivity at the interesting X-ray energies between 20 and 30 keV which is currently being realized by different detector companies. In addition, a translation from 2D imaging to a 3D method will help to more accurately correct attenuation in different organs. First pilot studies of these advanced approaches have already been tested and reported in^24^.

The here presented measurements demonstrate that XFI can be a useful imaging modality which can be applied to answer different preclinical and, in the future, even clinical questions and has the potential to close the existing gap between already established imaging techniques. Besides its described applications in cell tracking within the context of immune-mediated inflammatory diseases, pharmacological studies in many different areas such as oncology could potentially profit from the modality. Further improvements include the development of an efficient multi-detectors setup as well as the translation to a 3D technique which both will allow to increase the detection sensitivity for future research questions. Finally, the use of a synchrotron X-ray source as done in this study is impractical for future clinical applications as those machines are huge, expensive, and only have very limited access. One potential solution for the translation into clinics is the use of compact, laser-driven X-ray sources which have become an active field of research in the past years^25–28^.

## Methods

### MHS cell culture

The mouse macrophage alveolar cell line MHS was obtained from American Type Culture Collection (ATCC, Manassas, VA, USA, Roswell Park Memorial Institute (RPMI)). 1640 medium with heat inactivated 10% fetal bovine serum (FBS), 1 mM Na-pyruvate, 100 U/ml penicillin, and 100 µg/ml streptomycin (Gibco Invitrogen Corporation) and β -mercaptoethanol (0.05 mM) was used to culture the cells at 37 °C in 5% CO_2_.

### Cell viability assay

**Figure 4:**
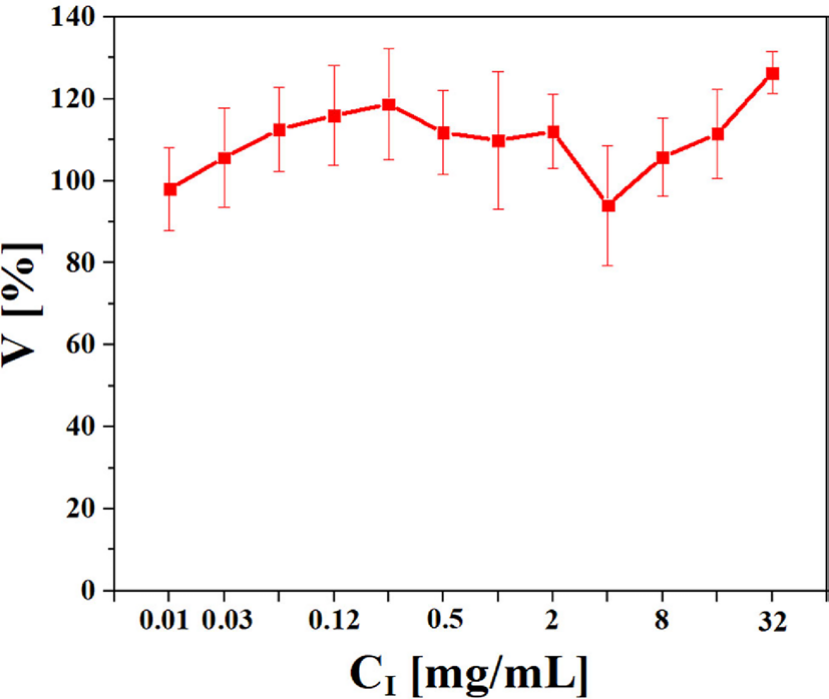
Cell viability V in % of MHS cells exposed to iohexol at different exposure concentrations, given as element concentrations of iodine, C_I_, after 24 h exposure as detected by the Resazurin assay. The results are presented as mean values $\pm$ standard deviations from three individual experiments.

In order to investigate the cell viability of MHS exposed to iohexol, a Resazurin assay was conducted as previously reported^29,30^. Briefly, 3.3 × 10^4^ cells in 100 µl medium were seeded in 96 well plates and cultured overnight. The next day, different concentrations of iohexol were added and cells were again incubated for 24 h. One iohexol molecule contains three iodine atoms. In the following, the iohexol concentration is given in terms of mass concentration of iodine, C_I_. After incubation with iohexol, cells were washed one time with phosphate buffer saline (100 µl per well). Subsequently, Alamar Blue solution (Thermal Fisher scientific) was added into the wells according to the instructions of the supplier and cells were incubated for another 4 h at 37 °C. Afterwards, the fluorescence from each well was detected with a microplate reader (Jobin Yvon Fluorolog-3 with microplate reader adapter) from 580 to 590 nm. The mean detected fluorescence intensity was considered to be linear to the cell viability. For normalization to 100% cell viability the mean fluorescence intensity of cells without iohexol exposure was used^31^ and the results are presented in Fig. 4.

### Cell proliferation assay

**Figure 5:**
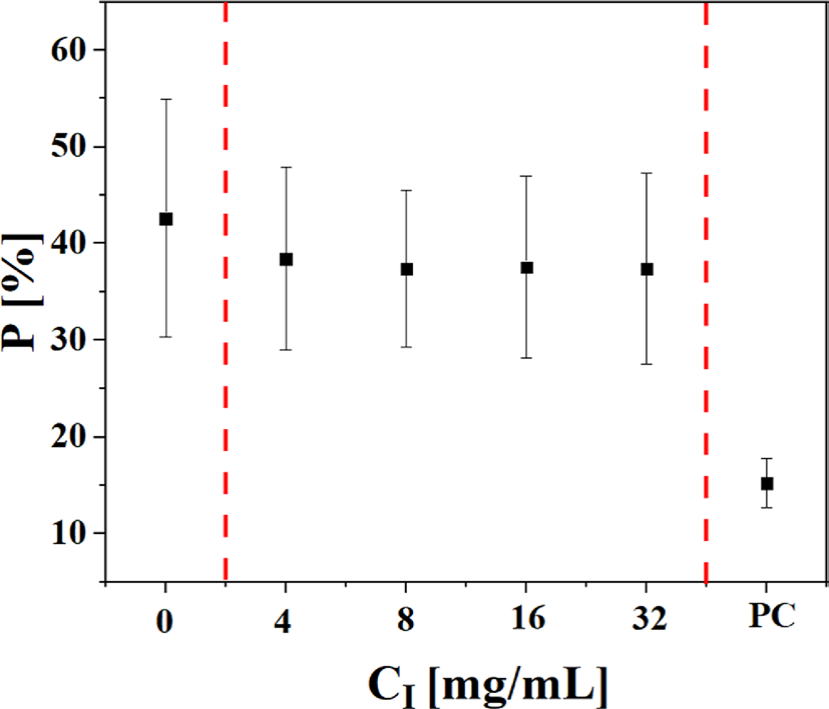
Cell proliferation P of MHS cells after 24 h exposure to iohexol at different elemental concentrations of iodine, C_I_. Colchicine was chosen as positive control (PC) and cells exposed to medium only (i.e., C_I_ = 0) were the negative control. The results are presented as percent proliferation P [%] (mean) from three individual experiments.

In order to study whether cell proliferation was influenced after iohexol exposure, the assay described in the following was carried out^31^. 3.3 × 10^4^ cells in 100 µl medium were seeded in 96 well plates and were incubated overnight. The next day, the medium was replaced by fresh medium containing different concentrations of iohexol, and cells were incubated for another 24 h at 37 °C and 5% CO_2_. Colchicine (5 µM) was added instead of iohexol as positive control to suppress cell proliferation. As negative control, no iohexol was added. After 24 h exposure the medium was replaced by fresh medium containing ethynyllabeled deoxyuridine (EdU, 5 µM) and cells were incubated for another 6 h. The medium was then removed and 100 µl of 3.7% formaldehyde was added into each well to fix the cells, followed by 15 min incubation at room temperature. After that, the fixative was removed and 3% bovine serum albumin (BSA) in phosphate buffered saline (PBS, 100 µl) was used to wash the cells twice. Afterwards, 100 µl of 0.5% Triton X-100 in PBS was added into each well. After incubation for 20 min at room temperature the permeabilization buffer was removed and cells were washed twice with 3% BSA in PBS (100 µl). For labelling with Alexa Fluor azide 488 (Invitrogen Click-iT EdU Imaging Kit) a reaction solution of Alexa Fluor azide and ascorbic acid in tris(hydroxymethyl)aminomethane (Tris) buffer at pH = 8.5, 100 mM CuSO_4_ was prepared according to the manual of the vendor. Then, 50 µl of reaction solution were added to each well and incubated for 30 min protected from light. After that, the reaction solution was removed, followed by washing once with 3% BSA in PBS (100 µl). After one more washing step with 100 µl PBS, 50 µl Hoechst (5 µg/ml) in PBS were added to stain the nuclei for 30 min at room temperature. Cells in each well were then washed twice with 100 µl PBS. Finally, 100 µl PBS was added to each well before imaging of the cells by confocal microscopy. To determine the proliferation rate, the images were processed by CellProfiler and the Alexa Fluor fluorescence per cell was determined. This fluorescence is considered to be proportional to synthesized DNA upon proliferation. The fluorescence of cells without exposure was defined as proliferation rate of P = 100%. Finally, the proliferated cells from all cells were calculated as the cell proliferation P for each image and averaged for each concentration as shown in Fig. 5.

### Cell migration assay

**Figure 6:**
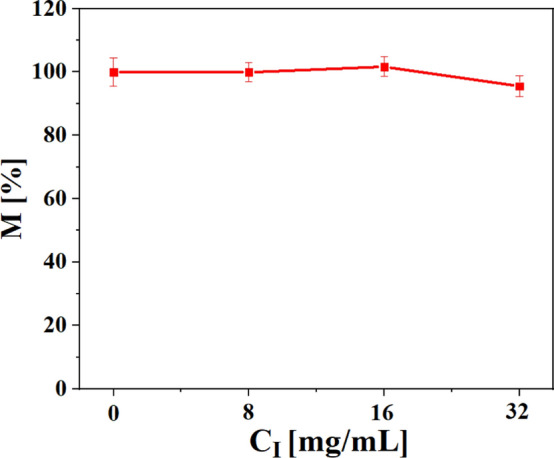
Cell migration assay of MHS cells after 24 h exposure to iohexol at different elemental concentrations of iodine, C_I_. The results are presented as percent of migration M [%] and the mean values $\pm$ standard deviations from three individual experiments are displayed.

In order to study whether the migration ability of cells exposed to iohexol was affected, a cell migration assay was conducted, following an adopted protocol^32^. Briefly, 10^6^ MHS cells were seeded per 6 well plate, followed by incubation overnight. The next day, the cell medium was replaced by fresh cell medium supplemented with different concentrations of iohexol, and cells were incubated for 24 h at 37 °C and 5% CO_2_. The cells were then detached from the wells by addition of trypsin- ethylenediaminetetraacetic acid (EDTA) and cells were transferred into FBS supplemented cell medium. Migration chambers (8 µm pore size, Greiner Bio One, #662638) were put into 24 well plates. First, 100 µl serum-free medium was added into each chamber. Then, 200 µl cell suspension (3 × 10^4^ cells) was placed into the upper part of each chamber and 800 µl FBS supplemented cell medium was placed into each well, followed by 16 h incubation at 37 °C and 5% CO_2_. After incubation the medium from the chambers as well as the chamber itself were washed with PBS twice. Then, 3.7% formaldehyde in PBS was used to fix the cells for 10 min at room temperature. Afterwards, the cells were washed twice with PBS and the inner side the of chamber was wiped by a swab to remove the cells which had not migrated through the membrane of the migration chamber. Finally, the chamber was placed on a microscope plate and was imaged by bright field microscopy. Cell images were analyzed by the software ImageJ to count the number of cells which had migrated to the backside of each chamber. The number of migrated cells was divided by the number of seeded cells and multiplied by 100 to provide the percentage of cells which had migrated through the chamber as presented in Fig. 6.

### Cellular uptake of iohexol

**Figure 7:**
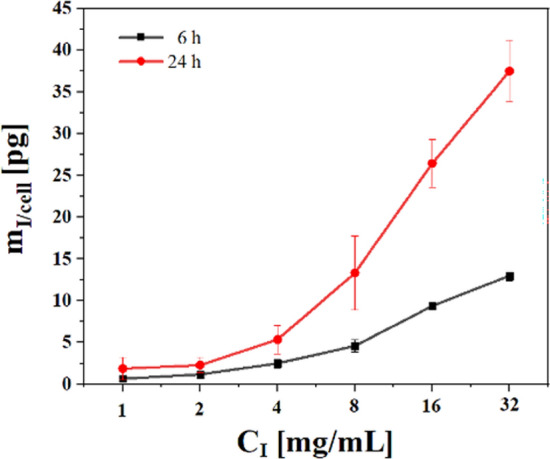
Amount of elemental iodine found per cell in MHS exposed to iohexol at different exposure concentrations after 6 and 24 h incubation as determined by ICP-MS. The results are presented as mean values ± standard deviations from three individual experiments.

The labelling efficiency of MHS with iohexol was evaluated by ICP-MS as previously reported^29,30^. Briefly, 10^6^ cells in 2 ml medium were seeded in 6 well plates and incubated overnight at 37 °C and 5% CO_2_. The next day, the medium was exchanged and different iohexol concentrations were added. Cells were then incubated for 6 h or 24 h. After incubation, the supernatant was discarded and each well was washed three times by PBS. Then, 100 µl trypsin ethylenediaminetetraacetic acid (EDTA) (0.01% trypsin–EDTA, Thermo Fisher Scientific) were added into each well for 1 min to detach the cells. 1 ml cell medium was added to collect all cells and the cell suspension was centrifuged at 300 rcf for 5 min. The resulting cell pellet was dissolved in 1 ml PBS and the number of cells was counted. With ICP-MS the amount of iodine in the cell pellet was determined. Dividing by the number of cells in the pellet yields the mass of iodine per cell, m_I/cell_, as presented in Fig. 7.

### Exocytosis of iohexol

**Figure 8:**
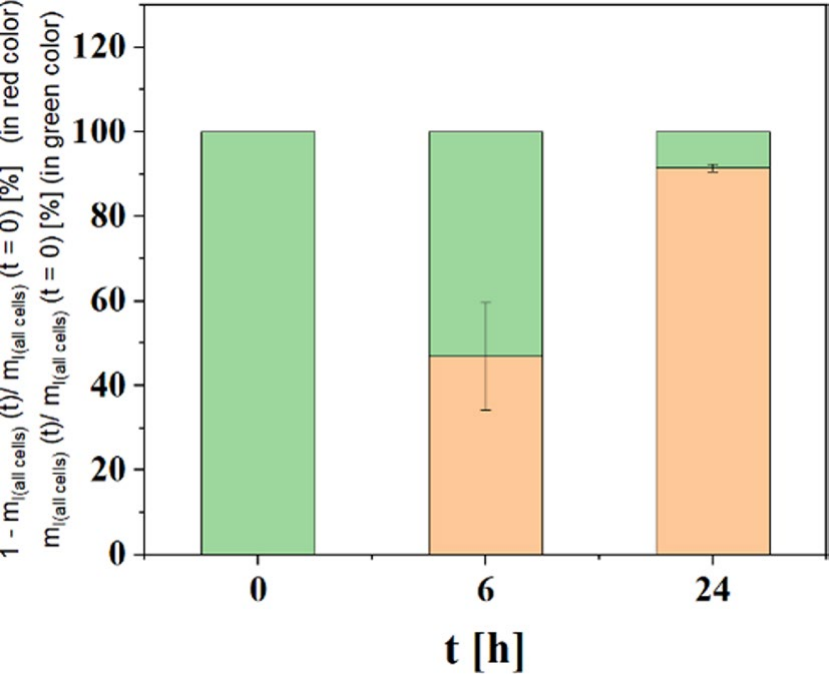
Fraction of iohexol which after time t has been exocytosed (1 − m_I(all cells)_ (t)/m_I(all cells)_ (t = 0)) shown in red, and fraction of iohexol which after time t remains internalized by cells (m_I(all cells)_ (t)/m_I(all cells)_ (t = 0)) shown in green. The data shown corresponds to the mean value from three experiments.

In order to study the retention of iohexol in cells after endocytosis, exocytosis of iohexol was investigated according to a previously published protocol^29,30^. Briefly, 10^6^ MHS in 2 ml cell medium were seeded per well in 6 well plates and were incubated overnight at 37 °C and 5% CO_2_. Then the medium was replaced by 2 ml fresh cell medium supplemented with iohexol. Iohexol exposure at a final concentration of C_I_ = 16 mg/ml was carried for t = 24 h. After that, the medium was removed and cells were washed with PBS for 3 times in order to remove non-internalized iohexol. Subsequently, 2 ml new cell medium was added into each well and cells were incubated for t = 0, 6, and 24 h. After the desired incubation time, the cells were collected by trypsination and the total amount of iodine in all cells, m_I(all cells)_, was determined by ICP-MS. Note that upon passing iohexol to daughter cells upon proliferation the total amount of iodine in all cells is not affected. However, upon exocytosis the total amount of iodine in all cells is reduced over time^33^. The percentage of exocytosed iohexol was calculated as 1 − m_I(all cells)_ (t)/m_I(all cells)_ (t = 0). The percentage of iohexol remaining inside cells is m_I(all cells)_ (t)/m_I(all cells)_ (t = 0), as shown in Fig. 8.

### Experimental setup and data taking

The measurements were taken at the P21.1 beamline at the PETRA III synchrotron at DESY, Hamburg, Germany, where an incident energy of about 53 keV is available. As motivated above, this energy is a good compromise between background reduction on the one hand and fluorescence cross-section on the other hand. The unattenuated photon flux in a 1.0 × 1.0 mm^2^ spot was 8 × 10^10^ photons/s at the provided energy as determined by measurements of several reference targets purchased from Micromatter (Surrey, BC, Canada). The deposited layers contain different fluorescence elements of defined masses per area which allows a precise reconstruction of the incident photon flux and hence quantification of results. Different beam sizes can be chosen with a pair of slits and attenuation of the incident beam flux is possible with an absorber system. For continuous flux monitoring, silicon PIN diodes are installed before and after the scan object to allow correction of possible fluctuations. The local energy dose per scan position was below 300 mGy, which is tolerable as T cells can repair such low-dose induced beam damages^14,15^. The main experimental parameters used for the presented measurements as well as the local energy dose are summarized in Table 1.

**Table 1:** Summary of the main scan and mouse parameters used for the in vivo measurements.

Injection	Scan time/pixel [s]	Dose [mGy]	Time [h]
200 µl of 100 mg/ml iohexol	0.5	130	6/17.5
10^7^ MHS with 16 mg/ml iohexol	0.75	190	6
10^7^ MHS with 16 mg/ml iohexol	0.75	190	6

Note that mouse 1 was measured twice in order to determine the biodistribution of the injected contrast agent iohexol over time. Also note that the local energy dose levels are well below the 300 mGy-level.

After anesthesia, the mice were positioned in a commercially available imaging cell from Minerve (Equipement Veterinaire MINERVE, Esternay, France) on a custom-made holder to mount the cell on a translation arm offering three degrees of freedom. For fluorescence signal detection a silicon drift detector (SDD) of 50 mm^2^ collimated area and 0.5 mm sensor thickness from Amptek (X-123FASTSDD, Amptek Inc, MA, USA) was positioned at 57 mm distance from the center of the mouse under a detection angle of 150 degrees with respect to the incident beam axis.

**Figure 9:**
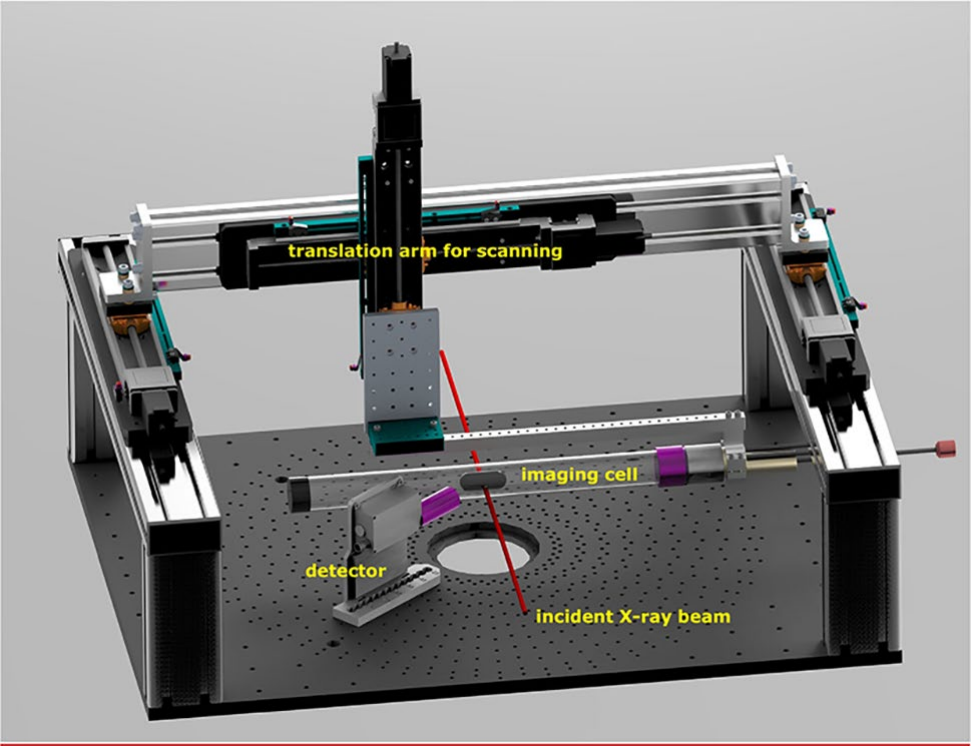
Schematic of the experimental setup used at the P21.1 beamline at the PETRA III synchrotron, consisting of a dedicated holder for the imaging cell, an X-ray detector and a custom-made translation arm to allow scanning of the mice.

Figure 9 shows a schematic of the experimental setup, mainly consisting of a custom-made imaging platform on which the translation arm with a dedicated holder for the imaging cell as well as one or several radiation detectors can be positioned. This platform can be mounted on a diffractometer in the experimental hutch and the incident X-ray beam is indicated in red.

Even though SDDs offer a lower efficiency in the iodine fluorescence energy region around 30 keV, their energy resolution as well as count rate capability are superior to high-Z semiconductor detector materials such as CdTe and Ge. In addition, a 0.5 mm thin sensor allows for reduction of the total scan times as its efficiency at 44 keV, where the majority of photons from Compton scattering is registered, is only 4%, while it still offers around 14% in the iodine K_*α*_ signal region. Note that all data presented here was only taken with a single X-ray detector which is currently extended to a multi-detectors setup to cover a significantly larger solid angle. In order to keep scanning times short, a continuous mode was implemented in the used detector readout software. Motor movements typically significantly contribute to the total duration of a measurement and hence also the applied dose when no beam shutter is used between the single steps. In the chosen continuous scan mode, the horizontal axis is translated continuously and consequently, X-ray spectra are recorded on the spur of the movement, allowing much faster scan times of about 18 min per full-body scan. The beam size in this mode was 0.1 × 1.0 mm^2^ with the short side along the horizontal axis to minimize blurring between adjacent data points. In this configuration, spectra are recorded over a travel length of 1 mm where the scan duration per pixel is controlled by the motor travel speed. In contrast, in the step-scanning mode, motors are moved to their dedicated positions and only after the acquisition has finished, can be moved to the next position. Since typical motor accelerations are limited in order to prevent vibrations, an additional time overhead is created which makes total scan durations significantly longer and hence this mode is not suited for in vivo measurements.

### Data analysis and mass reconstruction

Peak fitting was performed in order to accurately determine the amount of iodine fluorescence photons as described in^13^. From those signal counts, the iodine mass per scan point area can be determined by applying an average distribution model for correcting for the signal photon attenuation. This model calculates the average attenuation over 8 different distributions of the iodine mass along the X-ray beam volume. A recent development of a 3-dimensional version of our advanced XFI-approach shows that this average distribution model is a good approximation for the attenuation correction, as presented in^24^. For mouse-sized objects the attenuation of both the incident photons and the excited outgoing X-ray fluorescence photons is not problematic: we deliberately use an incident energy of 53 keV, thus way above the K-absorption edge of iodine, and even the X-ray fluorescence photons can still effectively traverse the object, which is a clear advantage of XFI over optical fluorescence. In this sense, there is no effective depth limit for mouse-sized objects as the corresponding transmission is typically on the order of 60%.

### Multi-tracking XFI data

XFI has the advantage of providing the simultaneous tracking of different marker elements in one and the same scan. Thus, whenever there are different markers inside the scanning beam volume, all of them emit their specific X-ray fluorescence energies which all appear in the spectrum measured by an X-ray detector. There is no strict limit on the choice of suitable elements, but if they are close-by in the Periodic Table (e.g., iodine and palladium), their physical properties (like fluorescence cross-section and attenuation of their fluorescence photons) are also similar. However, it is useful to choose such elements whose X-ray fluorescence lines are in that part of the recorded spectra in which the spectral background is minimal, as explained above.

**Figure 10:**
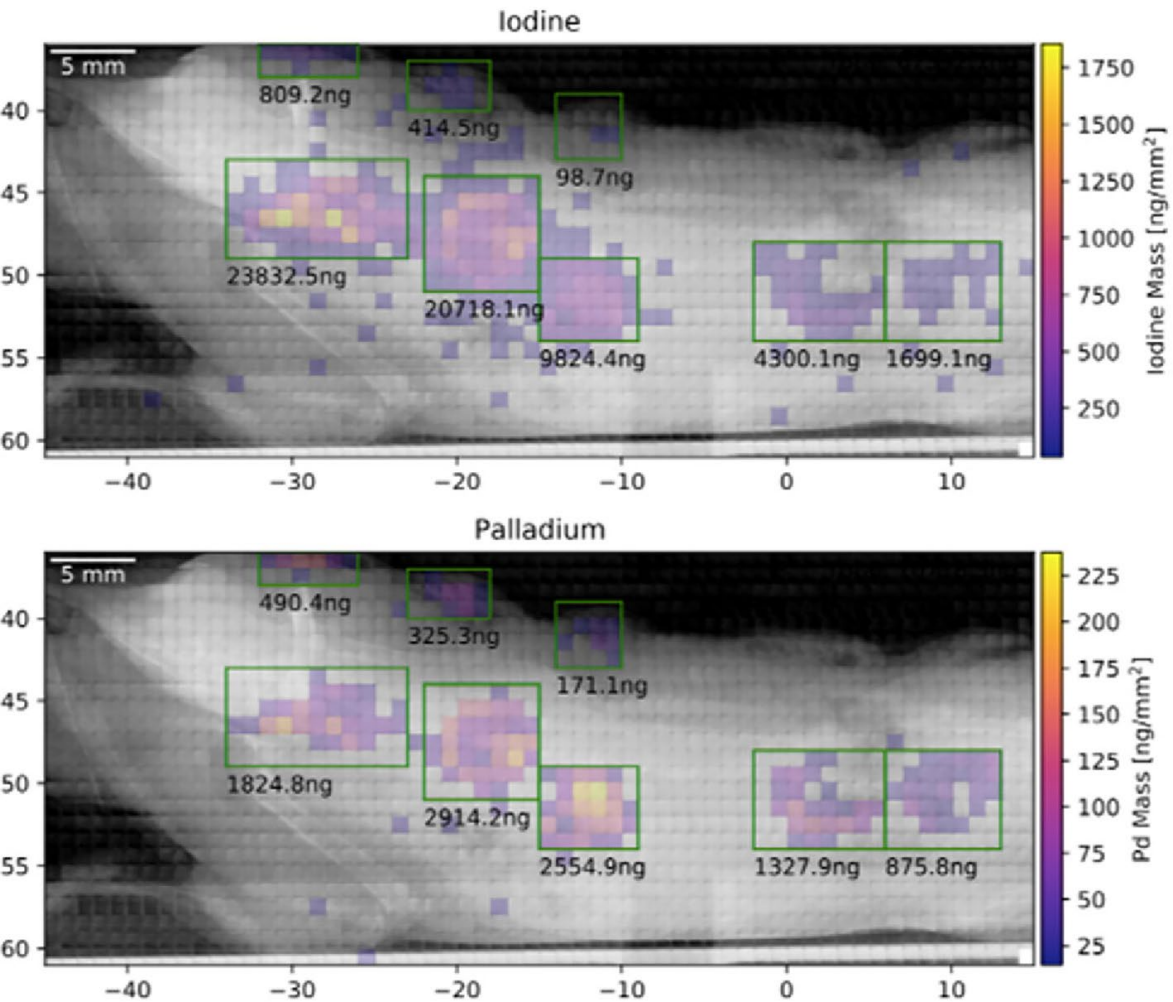
XFI-maps of the two different markers (molecular iodine on top, palladium-nanoparticles at the bottom) from the eight injection sites of one and the same mouse.

**Figure 11:**
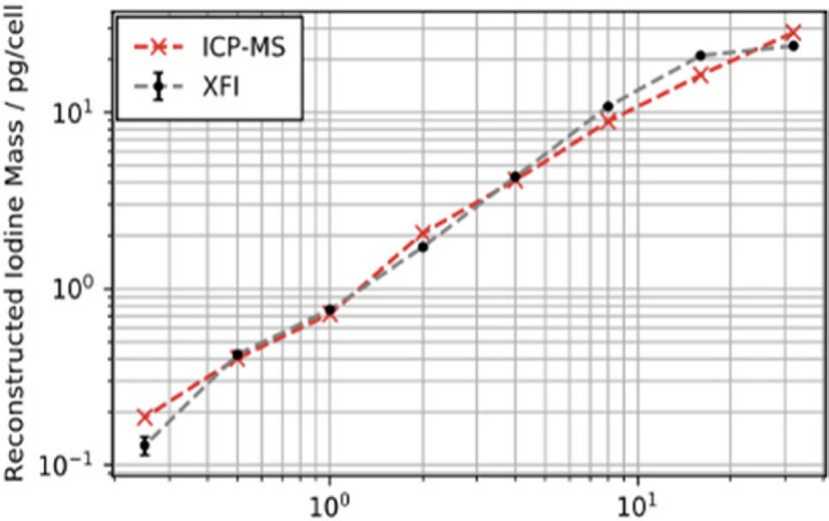
The direct comparison between the marker’s masses, given in units of mass per cell, between XFI and ICP-MS shows very good agreement with a mean and standard deviation of the ratio between ICP-MS and XFI as 1.03 $\pm$ 0.21 over all 8 data points. This underlines both the multi-tracking capability of XFI as well as its quantitative character.

In order to demonstrate this multi-tracking, we have chosen to use two different types of markers: 50% of a macrophage sample to be injected into the back of a dead mouse was labelled with iohexol, while the other 50% were marked with palladium (Pd)-nanoparticles. Figure 10 shows the XFI-map of the iodine signal as well as the Pd-map. Note that both maps stem from the same mouse scan. One can clearly see the eight injection sites, whereby the respective marker concentrations were varied from site to site. Also shown in Fig. 11 is a direct comparison between the marker masses retrieved from the measured XFI-data (Fig. 10) and the ICP-MS measurement of a fraction of the above-mentioned sample, that is, from cells that have not been injected, but used for the ICP-MS analysis. The agreement is very good over almost more than two orders of magnitude.

### Animal studies

9–13 weeks old C57BL/6 Rag1^−/−^ mice were used as recipients. Mouse 1 received 200 µl of 100 mg/ml iohexol in saline solution intraperitoneally (i.p.) injected.

Mice 2 and 3 were injected i.p. with 10 million macrophages labelled with 16 mg/ml iohexol. During imaging, mice were anesthetized with Isofluoran (Baxter GmbH, Germany). All mice were cared for in accordance with German animal ethics regulations and experimental protocols were approved by the institutional review board Behörde Justiz und Verbraucherschutz, Hamburg, Germany (N 024 2020 and ORG 998). The experiments were performed in accordance with the ARRIVE guidelines.

## Data Availability

The datasets used and/or analyzed during the current study are available from the corresponding author on reasonable request.

## Code availability

Code will be made available upon reasonable request.
